# Mediating effects of insulin resistance on lipid metabolism with elevated paraben exposure in the general Taiwan population

**DOI:** 10.3389/fpubh.2025.1416264

**Published:** 2025-03-14

**Authors:** Po-Chin Huang, Hsin-Chang Chen, Han-Bin Huang, Yu-Lung Lin, Wan-Ting Chang, Shih-Hao Leung, Hsi Chen, Jung-Wei Chang

**Affiliations:** ^1^National Institute of Environmental Health Sciences, National Health Research Institutes, Miaoli, Taiwan; ^2^Department of Medical Research, China Medical University Hospital, China Medical University, Taichung, Taiwan; ^3^Research Center for Precision Environmental Medicine, Kaohsiung Medical University, Kaohsiung, Taiwan; ^4^Department of Chemistry, Tunghai University, Taichung, Taiwan; ^5^School of Public Health, National Defense Medical Center, Taipei, Taiwan; ^6^Institute of Environmental and Occupational Health Sciences, School of Medicine, National Yang Ming Chiao Tung University, Taipei, Taiwan

**Keywords:** parabens, endocrine disruptors, lipid metabolism, insulin resistance, mediation analysis

## Abstract

**Introduction:**

Parabens are commonly used to prevent bacteria from growing in cosmetics and foodstuffs. Parabens have been reported to influence hormone regulation, potentially leading to metabolic anomalies, including insulin resistance and obesity. However, there is a paucity of knowledge regarding the relationship between urinary paraben levels and lipid metabolism in the general Taiwanese population. Therefore, the objective of this study was to determine whether the mediating role of insulin resistance exists between paraben exposure and lipid metabolism.

**Methods:**

We selected the data of 264 adult participants from a representative survey in five major Taiwan area in 2013. UPLC tandem mass spectrometry was used to examine four urine parabens: methyl- (MeP), ethyl- (EtP), propyl- (PrP) and butyl- (BuP). Blood samples were analyzed for concentrations of glucose and lipid metabolic indices using the DxI 800 immunoassay analyzer and immunoradiometric assay kit. The relationship between urinary paraben levels and metabolism indices were evaluated through a multiple linear regression analysis. Finally, a mediation analysis was employed to understand the underlying mechanism by which paraben exposure influences lipid metabolism through insulin resistance.

**Results:**

The significant positive association between MeP exposure and Castelli risk index I (CRI-I; β = 0.05, *p* = 0.049) was found, and also exhibited the similar associations between EtP exposure and low-density lipoprotein cholesterol (β = 0.10, *p* = 0.001), total cholesterol (β = 0.06, *p* = 0.003), and non-HDL cholesterol (NHC; β = 0.08, *p* = 0.005). EtP exhibited a significant positive association with triglyceride BMI (TyG-BMI; β = 0.02, *p* = 0.040). Additionally, TyG-BMI was positively associated with CRI-I (β = 0.98, *p* < 0.001), CRI-II (β = 1.03, *p* < 0.001) and NHC (β = 0.63, *p* < 0.001). Moreover, insulin resistance served as mediators for the effects of EtP exposure on lipid metabolism indices.

**Discussion:**

The results indicate that changes in insulin resistance mediated the relationship between urinary paraben and lipid metabolism. Large-scale epidemiological and animal studies are warranted to identify biological mechanisms underlying validate these relationships.

## 1 Introduction

Obesity has reached epidemic proportions globally, imposing a considerable public health burden in both developed and developing countries (1). According to the Nutrition and Health Survey in Taiwan (NAHSIT), the prevalence of general and abdominal obesity has been increasing from 16–20% and 27–47% from 1993–2016, respectively (55), and it increases the risk of chronic diseases such as cardiovascular disease, and type 2 diabetes (2). In Taiwan, the average body mass index (BMI) of adults is 24.5 kg/m^2^, which exceeds the standard for overweight set by Taiwan’s Health Promotion Administration (BMI ≥ 24 kg/m^2^). The prevalence of overweight and obesity in adults was reported to be 50.7%, indicating that approximately half of the adult population has obesity (56). Factors such as genetic predisposition and lifestyle choices, including diet and physical activity, contribute to the complex etiology of obesity (3, 4). There is more evidence that certain endocrine disruptors, such as parabens, could play a role in the development of obesity and diabetes (5–7).

Parabens are extensively used as artificial preservatives in cosmetics and foodstuffs (8, 9) due to their chemical stability, low cost, and broad-spectrum antimicrobial properties (10). The chemical structure of parabens comprises a benzene ring, with a hydroxyl group and an ester group on the para position (11). Parabens differ in the alkyl chain length on the ester group and can be categorized into two types, including short alkyl-chain parabens (e.g., methylparaben, MeP) and long alkyl-chain parabens (e.g., butylparaben, BuP) (9). In addition, methyl paraben (MeP), ethyl paraben (EtP), propyl paraben (PrP), and butyl paraben (BuP) are the most commonly used parabens in commercial products (12), with the maximum usage level of 0.4% for a single compound and 0.8% for mixtures (13). Moreover, benzylparaben (BzP) and heptyl paraben (HeP) were not always incorporated into exposure assessments and statistical analyses due to their low frequency of detection in previous studies, unless stated otherwise (14).

Typical exposure routes for parabens include inhalation, ingestion, and dermal absorption; the latter is the primary exposure route for the general population, primarily owing to the widespread use of parabens in Personal care products (PCPs) (10). In recent years, the potential of parabens to cause endocrine disruption has elevated concerns about exposure to these chemicals.

*In vitro* studies have found that parabens have abilities in activating the glucocorticoid receptor and peroxisome proliferator-activated receptor *γ* (PPARγ) in 3 T3-L1 preadipocytes. Parabens could promote the differentiation of 3 T3-L1 adipocytes and increase their adipogenic potency, such as by increasing the synthesis and accumulation of triglycerides (15). Furthermore, animal studies have indicated that parabens can stimulate adipocyte differentiation and lipogenesis in white adipose tissue and liver fat cells in female rats, which includes the processes of fatty acid synthesis and subsequent triglyceride synthesis (16).

In epidemiology, studies have also evaluated associations between paraben exposure and human lipid-related traits. Parabens can cause a range of adverse health effects, particularly in the endocrine system. A growing body of evidence from epidemiological and toxicological studies indicates that paraben exposure may be associated with metabolic disorders, including obesity and diabetes mellitus (DM). A longitudinal study conducted on a sub-sample of the Granada EPIC-Spain cohort (*n* = 670) revealed that individuals with elevated levels of PrP exhibited an elevated risk of developing type 2 diabetes after a 23-year follow-up period (57). Furthermore, exposure to MeP and EtP has been linked to an increased risk of DM, with EtP exhibiting a positive association with a higher risk of obesity (17). Blood plasma samples were collected from 27 healthy women at various points throughout their menstrual cycles in order to examine the potential correlation between paraben exposure and obesity (58). The plasma levels of methylparaben, as well as the sum of parabens, were found to be positively associated with plasma adipsin levels. Conversely, a negative correlation was observed between methylparaben levels and glucagon, leptin, and PAI-1.

Some critical indices for assessing lipid metabolism include Triglyceride (TG), Total cholesterol (TC), high-density lipoprotein cholesterol (HDLC), and low-density lipoprotein cholesterol (LDLC). Additionally, other indices such as Castelli risk index (CRI-I and CRI-II), non-HDLC (NHC), and the atherogenic coefficient (AC) have been used to assess cardiovascular disease status and coronary artery disease risk (18–20). Disruptions in glucose homeostasis could also affect lipid metabolism. Moreover, insulin resistance can alter systemic lipid metabolism, leading to dyslipidemia. It can lead to elevated TG and LDLC levels and reduced HDLC levels (21, 22).

The above studies suggest that endocrine disrupters may affect human lipid metabolism. Moreover, the literature also reveals that the Taiwanese is commonly exposed to parabens (23, 24). However, the knowledge gap that exists between paraben exposure and lipid metabolism in the general Taiwanese population. Furthermore, fewer studies have investigated the mechanism through which insulin resistance mediates the relationship between urinary paraben levels and lipid metabolism.

To address the aforementioned research gaps, we aimed to investigate the relationship between urinary paraben levels, insulin resistance, and lipid metabolism in Taiwanese adults. Additionally, we explored whether insulin resistance served as mediators for the effects of paraben exposure on lipid metabolism indices. It is hypothesized that parabens may contribute to the development of obesity and cardiovascular disease through the promotion of insulin resistance and dyslipidaemia.

## 2 Methods

### 2.1 Characteristics of participants

Participants for this study were selected from the Taiwan Environmental Survey for Toxicants (TEST) 2013. A number of studies have previously detailed the participant recruitment, selection methods and approval from the Institutional Review Board of National Yang Ming Chiao Tung University in Taiwan (23–25). For the present study, participants aged ≥18 years were included and selected from 11 counties and cities from 5 regions in Taiwan: Northern, Central, Southern, Eastern, and one remote island. The sampling period spanned from May to December 2013. A total of 394 individuals were included through events held at elementary schools and community centers, yielding a response rate of approximately 78%. Before enrollment, all individuals provided informed consent and volunteered to participate in the NAHSIT. After the participants provided informed consent, their first-morning urine samples and fasting blood samples were collected; The concentration of parabens in the urine of an individual may fluctuate considerably over time due to a number of factors, including age, sex, lifestyle, diet, medical history, and environmental exposures. The precise impact of these variables on urinary paraben exposure among study participants remains unclear. Each participant was requested to complete a retrospective questionnaire encompassing demographic information (age and sex), BMI (body mass index) categories (i.e., <24 kg/m^2^, 24 < = & <27 kg/m^2^, and ≥ 27 kg/m^2^), geographical location (northern, central, southern, eastern, and remote islands), and educational attainment (≤elementary school, junior high school, senior high school, and ≥ college/graduates), annual family income (<15,625, 15,625–31,250, >31,250 USD), lifestyle factors (cigarette smoking and alcohol consumption) and PCP uses. Participants were categorized into different groups for the purpose of comparison, including different age groups (18–40, 40–65, and 65 and older). In addition, the term “cigarette smoking” is defined as the act of consuming at least one cigarette per day, as reported by the subjects. The subjects were self-reported lifelong non-smokers (never-smokers) who had involuntarily inhaled smoke from cigarettes or other tobacco products. The term “alcohol consumption” is defined as the ingestion of at least one bottle of alcohol per week. Subject who self-reported using at least one kind of PCPs (personal care products), including body wash, lotion, perfume, and nail polishes. The BMI standard for adults was divided into three groups: weight standard (BMI < 24 kg/m^2^), overweight (24≦BMI < 27 kg/m^2^) and obesity (BMI≧27 kg/m^2^) (59).

Anthropometric variables, including height, body weight, percentage body fat, and body mass index (BMI), were measured in accordance with standardized procedures outlined by Lohman et al. (60). Body height was measured with a portable stadiometer (model AD-6227R, manufactured by A&D Co., Ltd., Tokyo, Japan) to the nearest 0.1 cm. Body mass (0.1 kg) was evaluated by a bioelectrical impedance analyzer (model BC-418, manufactured by Tanita, Japan). BMI was calculated by dividing body weight (kg) by body height squared (m).

Of the original 394 subjects, a total of 28 were excluded from the study due to an insufficient number of urine samples and 27 were excluded due to an insufficient number of biochemical indicators. Additionally, 75 minors were excluded from the study. Our study included 264 TEST participants aged >18 years. Of these participants, 55 were excluded owing to inadequate urine or blood samples. Accordingly, a total of 264 participants were recruited in this study, comprising 125 men and 139 women. Participants aged between 40 and 65 years constituted the largest proportion of our study population (47.7%) (Table 1; Supplementary Figure S1). Moreover, 50.0% of the participants were of standard body weight, and 24.1 and 26.3% were overweight and obese, respectively. Regarding education level, 29.2% of the participants held a college degree or above. Furthermore, 58.1% of the participants reported having an annual household income of <NT$500,000. Approximately 75% of the participants were nonsmokers; however, nearly half of the participants were exposed to secondhand smoke. In addition, 74.6% of the participants reported using PCPs.

**Table 1:** Demographic characteristics of the study participants (*N* = 264).

Variables	Item	*N*	%	Mean ± SD
Gender	Male	125	47.3	
	Female	139	52.7	
Age (years, mean ± SD)	All	264		53.5 ± 17.1
	18–40	62	23.5	
	40–65	126	47.7	
	65 and older	76	28.8	
BMI^a^ (kg/m^2^) (mean ± SD)	All	264		24.7 ± 4.38
	Normal	132	50.0	
	Overweight	64	24.2	
	Obese	68	25.8	
Region	Northern Taiwan	83	31.4	
	Central Taiwan	36	13.6	
	Southern Taiwan	73	27.7	
	Eastern Taiwan	45	17.0	
	Remote island	27	10.2	
Marriage status	Single	44	16.7	
	Married	193	73.1	
	Divorce/widowed	27	10.2	
Education	≤Elementary school	73	27.7	
	Junior high school	38	14.4	
	Senior high school	76	28.8	
	≥College/graduates	77	29.2	
Annual family income^b^ (NTD)	<15,625	147	58.1	
	15,625–31,250	69	27.3	
	>31,250	37	14.6	
Cigarette smoking^c^	Yes/No	64/200	24.2/75.8	
Passive smoker^d^	Yes/No	132/132	50.0/50.0	
Alcohol consumption^e^	Yes/No	34/226	13.1/86.9	
PCPs usage^f^	Yes/No	197/67	74.6/25.4	

a BMI standard for adults: weight standard (BMI < 24 kg/m^2^), overweight (24≦BMI < 27 kg/m^2^) and obesity (BMI≧27 kg/m^2^) (59).

b The currency exchange rate of converting USD to new Taiwan dollar (NTD) is 1:32.

c Subjects who self-reported consuming at least one cigarette per day.

d Subject who self-reported as lifelong nonsmokers (never-smokers) but involuntary inhalation of smoke from cigarettes or other tobacco.

e Subject consuming at least one bottle of alcohol drink per week.

f Subject who self-reported using at least one kind of PCPs (personal care products), including body wash, lotion, perfume, and nail polishes.

### 2.2 Paraben analysis

Parabens and their metabolites do not accumulate in the body, and are eliminated within a few hours of exposure (26, 27). Serum paraben concentrations, even after intravenous injection, decline quickly and remain low in the blood (28). Given the short half-life of parabens in blood, the parent compounds and their metabolites are conjugated and excreted in urine. Therefore, urinary measurements in humans can be used to estimate paraben uptake (29). Urinary levels of parent parabens can be used as biomarkers of recent human monitoring (30–33). Significant positive correlations between urinary and blood levels were also observed in a Chinese study, suggesting that urinary concentrations are good predictors of human exposure to parabens and metabolites (34). In the present study, the reagents and chemical standards as well as the measurement procedures for the four parabens used in our study are comprehensively described elsewhere (35). Briefly, spot urine samples were kept and stored at −80°C until analysis. For analysis, the collected urine samples were thawed at 4°C for 24 h. Each sample was extracted through a supported liquid extraction (SLE) column, and the extract was then eluted twice with 0.9 mL of dichloromethane. Finally, the extract was dried under vacuum conditions, followed by the addition of MeOH and Milli-Q water (both 100 μL) were added to reconstitute the extract for injection. Paraben concentrations were measured using a Waters Acquity UPLC system equipped with a Thermo Scientific™ Hypersil Gold™ column (50 × 2.1 mm, 1.9 μm) (35). The coefficient of determination for parabens (r^2^) was higher than 0.9952. We observed that the average recovery rates of the parabens at low, medium, and high concentrations were 91.6–100.9% (5.4–10.5%), 84.4–99.5% (1.9–7.1%), and 86.8–98.4% (1.7–13.7%), respectively. Furthermore, the within-run and between-run accuracy (>85%) and precision (<14.2%) of our measurements were noted to meet the standards set by the European Medicines Agency (36). In instances where the paraben concentrations fell below the LOD, the measured concentrations were substituted with half the LOD. The LOD and LLOQ of each paraben were evaluated by SLE using paraben-spiked artificial urine and were 0.1 and 0.3 ng/mL, respectively (23, 24, 35). The present study has revealed that parabens Urinary creatinine levels were measured by spectrophotometry using a picric acid reagent with a wavelength of 520 nm (DXC 800 Synchron; Coulter, Brea, CA, United States).

### 2.3 Measurement of concentrations of metabolism indices

The UniCel DxI 800 Access Immunoassay System analyzer and an immunoradiometric assay kit (DIAsource, Louvain-la-Neuve, Belgium) were used to measure the concentrations of insulin resistance indices (e.g., glucose and insulin) and lipid metabolism indices (e.g., TG, HDLC, LDLC, and TC). The measurements were conducted randomly by technicians who were not aware of the metabolic status in Taiwan accredited laboratories (37, 38). Among our participants, 17.4 and 26.4% exhibited fasting blood glucose and insulin concentrations outside the reference range, respectively, and 13.6, 9.5, 29.9, and 37.9% exhibited TG, HDLC, LDLC, and TC levels outside the reference range, respectively. Furthermore, metabolic status was calculated using metabolism indices used in previous studies, including TG glucose-body mass index (TyG-BMI), CRI-I, CRI-II, NHC, and atherogenic coefficient (AC). TyG-BMI is an effective indicator for assessing insulin resistance Equation 1. TG metabolites affect the insulin sensitivity of adipose and muscle tissues and have been extensively studied for predicting diabetes. CRI-I, also known as the cardiac hazard ratio, reflects coronary plaque formation Equation 2. Moreover, CRI-II and AC are effective predictors of coronary artery disease risk Equations 3, 5. NHC is an indicator for predicting cardiovascular disease Equation 4. These indices can be calculated as follows (18–20, 39):


(1) $$TyG-BMI=Ln\left(\frac{TG\times \mathrm{glucose}\;}{2}\right)\times BMI$$

where glucose represents fasting glucose (mg/dL), and TG (mg/dL) and BMI (kg/m^2^) are already defined earlier (39).


(2) $$CRI-I=\frac{TC}{\mathrm{HDLC}}$$

(3) $$CRI-II=\frac{\mathrm{LDLC}}{\mathrm{HDLC}}$$

where TC (mg/dL), LDLC (mg/dL) and HDLC (mg/dL) are already defined earlier (20).


(4) NHC=TC−HDLC

where TC (mg/dL) and HDLC (mg/dL) are already defined earlier (19).


(5) $$\mathrm{AC}=\frac{\left(TC-\mathrm{HDLC}\right)}{\mathrm{HDLC}}$$

where TC (mg/dL) and HDLC (mg/dL) are already defined earlier (18).

### 2.4 Statistical methods

The medians and geometric means (GMs) of the concentrations of urinary parabens and lipid metabolism indices are first calculated. Subsequently, we used the Mann–Whitney U test to assess differences in the concentrations of parabens and lipid metabolism indices between the genders. The correlation between parabens and lipid metabolism indices was evaluated through a Spearman correlation analysis.

In this study, a multiple linear regression analysis was conducted; for this analysis, the measured concentrations of parabens and metabolism indices were subjected to a natural logarithm transformation to satisfy the normality assumptions via the Kolmogorov–Smirnov test. Moreover, age (continuous), sex (categorical), BMI (categorical), education (categorical), income (categorical) and use of PCPs (categorical) were selected as covariates; this selection was based on the findings of relevant studies (17, 40) and on whether the inclusion of any of these covariates would engender a > 10% change in the estimated coefficient. Additionally, we adjusted for endocrine disease status to minimize potential interference effects of endocrine diseases on our analysis results. We also adjusted for the metabolite di(2-ethylhexyl) phthalate, considering its association with lipid metabolism, as indicated in previous research (23–25). Directed Acyclic Graphs (DAGs) were utilised to investigate the potential role of confounding variables in the association between urinary paraben levels and lipid metabolism indicator (see Supplementary Figure S2). The minimum sufficient adjustment sets for estimating the total effect of urinary paraben levels on lipid metabolism indicator were determined to be age, sex, BMI, education, income and use of personal care products (PCPs). The directed acyclic graph (DAG) was constructed using a web-based tool (DAGitty^®^ version 3.1; 61). A mediation analysis was conducted using PROCESS v4.2 to explore the effect of insulin resistance on the relationship between parabens and lipid metabolism. In the mediation analysis, both indirect and direct effects were assessed, and the proportion of insulin resistance mediated the relationship between parabens and lipid metabolism was estimated (62). All data analyses were performed using SPSS software (version 24.0), and a *p*-value below 0.05 was considered statistically significant.

## 3 Results

### 3.1 Urinary concentrations of parabens and blood lipid metabolism indices

Table 2 presents the detection rate for the parabens as well as the medians and GMs of the concentrations of the parabens. The detection rate for the parabens was 100%. The parabens could be ordered as follows (in descending order) in terms of the GMs of their concentrations: MeP (383 μg/L), PrP (109 μg/L), EtP (39.5 μg/L), and BuP (6.35 μg/L). After stratifying our participants by gender, we observed that the GM of the concentrations of the parabens was higher in men than in women (MeP: 411 vs. 360 μg/L; EtP: 40.8 vs. 38.4 μg/L; PrP: 115 vs. 104 μg/L; BuP: 6.65 vs. 6.10 μg/L). However, the Mann–Whitney U test revealed no significant difference in urinary paraben concentrations between the genders.

**Table 2:** Distribution of parabens concentration (μg/L) in the general Taiwanese adult population by sex (*N* = 264).

Parabens	Group	DR (%)^a^	N	GM (95%CI)	Min	25th (95%CI)	50th (95%CI)	75th (95%CI)	95th (95%CI)	Max	*p*-value^b^
MeP	Adults	100	264	383 (356–412)	64.2	257 (225–277)	399 (360–456)	622 (542–690)	1,025 (936–1,103)	1,188	
	Men	100	125	411 (369–457)	90.4	266 (239–310)	419 (368–471)	654 (548–767)	1,059 (972–1,116)	1,134	0.152
	Women	100	139	360 (323–404)	64.2	234 (209–273)	376 (321–456)	615 (499–697)	1,015 (909–1,094)	1,188	
EtP	Adults	100	264	39.5 (36.7–42.6)	6.86	25.9 (23.8–28.0)	38.8 (35.1–43.5)	64.3 (56.9–76.7)	107 (99.3–112)	130	
	Men	100	125	40.8 (36.1–45.3)	6.86	25.9 (22.6–31.1)	40.5 (34.5–44.2)	74.6 (54.5–85.5)	110 (100–120)	130	0.484
	Women	100	139	38.4 (34.6–42.5)	7.16	25.5 (21.7–28.1)	37.9 (32.7–45.3)	60.7 (54.3–71.7)	107 (90.2–111)	112	
PrP	Adults	100	264	109 (102–116)	26.5	77.7 (67.4–82.1)	117 (105–124)	165 (149–180)	226 (217–239)	258	
	Men	100	125	115 (104–124)	26.5	80.5 (72.1–92.2)	115 (97.1–139)	179 (153–195)	228 (216–238)	253	0.134
	Women	100	139	104 (96–114)	31.2	65.6 (57.0–79.7)	117 (101–125)	156 (139–170)	225 (205–243)	258	
BuP	Adults	100	264	6.35 (5.98–6.77)	1.39	4.46 (4.18–4.92)	6.60 (6.00–7.36)	9.47 (8.93–10.0)	14.2 (13.4–15.0)	16.7	
	Men	100	125	6.65 (6.08–7.20)	1.40	4.84 (4.21–5.42)	6.84 (5.81–8.02)	9.60 (8.65–10.9)	14.8 (13.9–15.7)	15.8	0.305
	Women	100	139	6.10 (5.61–6.74)	1.39	4.34 (3.91–4.84)	6.54 (5.55–7.45)	9.47 (8.36–9.99)	13.4 (12.3–14.7)	16.7	

GM = Geometric mean.

a DR = Detection rate: number of urine sample with level of each paraben above detection limit/all analyzed urine samples.

b Comparison of urinary paraben levels between men and women using Mann–Whitney U test; ^***^ *p* < 0.001.

We also observed that the detection rate for all lipid metabolism indices was 100% (Table 3). The GMs of the concentrations of TG, LDLC, HDLC, and TC were 109, 110, 56.4, and 190 mg/dL, respectively. After stratifying our participants by gender, we observed that the GM of the concentration of TG was significantly higher in men than in women (125 vs. 96.0 mg/dL, *p* < 0.001). The GM of the concentration of LDLC was also higher in men than in women (112 vs. 107 mg/dL, *p* = 0.299). By contrast, the concentration of HDLC was significantly higher in women than in men (62.5 vs. 50.1 mg/dL, *p* < 0.001), and the concentration of TC was higher in women than in men (192 vs. 188 mg/dL, *p* = 0.334).

**Table 3:** Distribution of lipid metabolism indicators in the general Taiwanese adult population by sex (*N* = 264).

	Group	DR (%)^b^	N	GM (95%CI)	Min	25th (95%CI)	50th (95%CI)	75th (95%CI)	95th (95%CI)	Max	*p*-value^c^
TG (mg/dL)^a^	Adults	100	264	109 (101–117)	35.0	72.0 (66.0–76.5)	102 (94.0–111)	147 (132–164)	284 (258–361)	3,821	
	Men	100	125	125 (112–139)	35.0	79.5 (74,0–92.5)	119 (103–133)	184 (157–224)	383 (280–582)	1,512	<0.001^***^
	Women	100	139	96.0 (88.5–107)	35.0	66.8 (60.0–72.5)	93.5 (85.0–104)	126 (116–137)	221 (175–274)	3,821	
HDLC (mg/dL)^a^	Adults	100	264	56.4 (54.6–58.5)	23.2	46.5 (45.6–48.9)	56.3 (54.4–58.3)	68.7 (64.3–70.8)	85.1 (81.0–96.0)	118	
	Men	100	125	50.1 (47.7–52.6)	23.2	42.7 (40.8–45.2)	48.9 (46.3–53.3)	58.2 (55.8–62.2)	73.5 (69.9–105)	117	<0.001^***^
	Women	100	139	62.5 (60.2–65.2)	30.7	52.8 (50.7–55.9)	62.2 (59.8–64.4)	73.0 (70.7–77.1)	89.5 (83.1–110)	118	
LDLC (mg/dL)^a^	Adults	100	264	110 (105–113)	30.0	90.0 (85.5–95.0)	112 (109–116)	139 (128–145)	174 (166–189)	271	
	Men	100	125	112 (106–118)	40.0	94.0 (83.0–101)	112 (109–121)	144 (129–149)	175 (161–199)	271	0.299
	Women	100	139	107 (101–113)	30.0	88.8 (83.0–94.0)	112 (98.0–118)	134 (124–144)	173 (166–190)	199	
TC (mg/dL)^a^	Adults	100	264	190 (186–195)	92.0	168 (163–173)	190 (184–195)	220 (211–229)	263 (252–276)	493	
	Men	100	125	188 (181–195)	120	166 (155–174)	185 (180–195)	220 (205–230)	263 (246–284)	365	0.334
	Women	100	139	192 (185–200)	92.0	168 (162–178)	192 (185–198)	222 (209–234)	267 (248–286)	493	

GM = Geometric mean TG = Triglycerides, HDLC = High Density Lipoprotein Cholesterol, LDLC = Low Density Lipoprotein Cholesterol, and TC = Total cholesterol.

a The laboratory reference ranges of adults for TG, HDL-C, LDL-C, and TC were < 150 mg/dL, > 40 mg/dL, < 130 mg/dL, and < 200 mg/dL, respectively.

b DR = Detection rate: number of urine sample with each lipid metabolism indicators concentration above detection limit/all analyzed urine samples.

c Comparison of lipid metabolism indicators concentration between men and women using Mann–Whitney U test; ^*^p < 0.05; ^**^p < 0.01; ^***^ *p* < 0.001.

### 3.2 Associations of urinary parabens with lipid metabolism and insulin resistance indices

**Figure 1:**
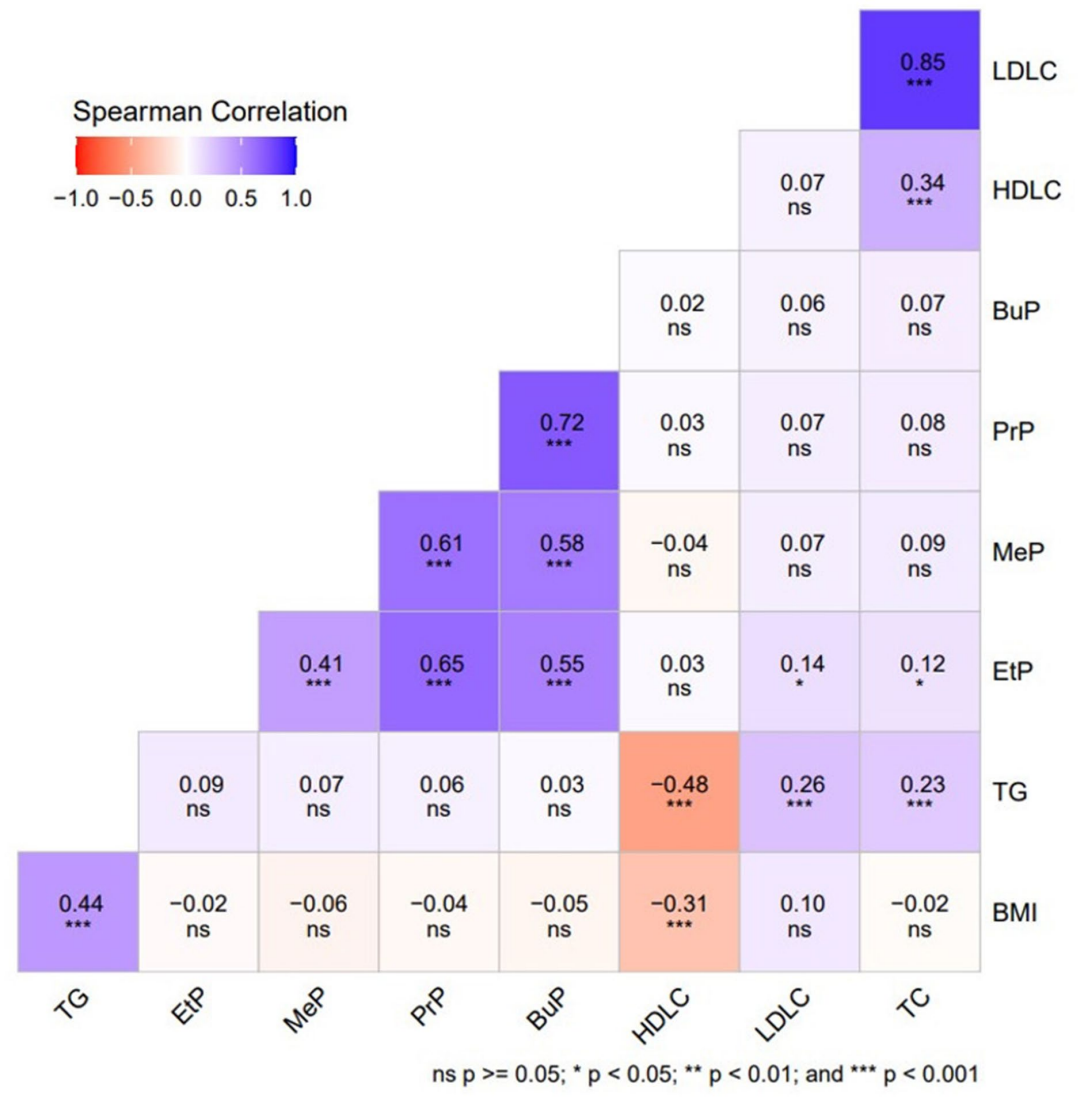
Spearman’s correlation coefficients between urinary paraben concentrations in adults and lipid metabolism indices (*N* = 264).

As indicated in Table 4 and Figure 1, our Spearman correlation analysis revealed a significant positive association between urinary parabens and lipid metabolism indices (*p* < 0.01). EtP was significantly positively correlated with LDLC (r = 0.139, *p* = 0.024) and TC (r = 0.123, *p* = 0.047).

**Table 4:** Spearman’s correlation coefficients between urinary paraben concentrations in adults and lipid metabolism indicators (*N* = 264).

	MeP	EtP	PrP	BuP	TG (mg/dL)	HDLC (mg/dL)	LDLC (mg/dL)	TC (mg/dL)	BMI (kg/m^2^)
MeP (μg/L)	1.000	**0.414** ^**^	**0.613** ^**^	**0.577** ^**^	0.068	−0.044	0.074	0.087	−0.062
EtP (μg/L)		1.000	**0.649** ^**^	**0.548** ^**^	0.090	0.027	**0.139** ^*^	**0.123** ^*^	−0.025
PrP (μg/L)			1.000	**0.719** ^**^	0.062	0.027	0.074	0.075	−0.037
BuP (μg/L)				1.000	0.028	0.025	0.056	0.070	−0.052
TG (mg/dL)					1.000	**−0.482** ^**^	**0.260** ^**^	**0.225** ^**^	**0.443** ^**^
HDLC (mg/dL)						1.000	0.066	**0.344** ^**^	**−0.314** ^**^
LDLC (mg/dL)							1.000	**0.849** ^**^	0.097
TC (mg/dL)								1.000	−0.023
BMI (kg/m^2^)									1.000

TG = Triglycerides, HDLC = High Density Lipoprotein Cholesterol, LDLC = Low Density Lipoprotein Cholesterol, and TC = Total cholesterol. ^*^p < 0.05; ^**^p < 0.01; ^***^ *p* < 0.001 Bold: *p* < 0.05.

The multiple linear regression model was also used to explore the association of urinary parabens with lipid metabolism indices and insulin resistance indices (Table 5). After controlling for confounders, we observed that MeP exhibited a significant positive association with CRI-I (β = 0.05, *p* = 0.049). EtP also exhibited significant positive associations with LDLC (β = 0.10, *p* = 0.001), TC (β = 0.06, *p* = 0.003), and NHC (β = 0.08, *p* = 0.005). Furthermore, regarding the associations between urinary parabens and insulin resistance indices, EtP was positively associated with TyG-BMI (β = 0.02, *p* = 0.040).

**Table 5:** Adjusted regression coefficients (β), 95% confidence intervals (CI), and *p*-values (*p*) for change in lipid and glucose metabolism indicators in relation to unit-increased in Ln-parabens (μg/L) in Taiwanese adults (*N* = 264).

Variable	MeP (μg/L)			EtP (μg/L)			PrP (μg/L)			BuP (μg/L)		
	β	95% CI	*p* value	β	95% CI	*P* value	β	95% CI	*P* value	β	95% CI	*P* value
Model 1^a^												
TG (mg/dL)	0.08	(−0.03, 0.18)	0.147	0.06	(−0.05, 0.17)	0.300	0.03	(−0.11, 0.16)	0.711	0.06	(−0.06, 0.19)	0.318
HDLC (mg/dL)	−0.03	(−0.07, 0.02)	0.250	0.03	(−0.02, 0.08)	0.192	0.03	(−0.03, 0.09)	0.306	0.01	(−0.04, 0.07)	0.684
LDLC (mg/dL)	0.03	(−0.03, 0.09)	0.340	**0.10**	**(0.04, 0.16)**	**0.001**	0.07	(−0.01, 0.15)	0.083	0.07	(<0.01, 0.14)	0.050
TC (mg/dL)	0.02	(−0.02, 0.06)	0.261	**0.06**	**(0.02, 0.10)**	**0.003**	0.04	(−0.01, 0.09)	0.134	0.04	(−0.01, 0.09)	0.086
CRI-I	**0.05**	**(<0.01, 0.10)**	**0.049**	0.03	(−0.02, 0.08)	0.229	0.01	(−0.06, 0.07)	0.788	0.03	(−0.03, 0.09)	0.312
CRI-II	0.06	(−0.01, 0.13)	0.115	0.07	(> − 0.01, 0.14)	0.059	0.04	(−0.05, 0.13)	0.410	0.06	(−0.02, 0.14)	0.155
NHC	0.04	(−0.01, 0.10)	0.123	**0.08**	**(0.02, 0.13)**	**0.005**	0.04	(−0.03, 0.11)	0.230	0.06	(−0.01, 0.12)	0.096
AC	0.07	(> − 0.01, 0.14)	0.057	0.05	(−0.03, 0.12)	0.205	0.01	(−0.08, 0.11)	0.793	0.04	(−0.04, 0.13)	0.316
TyG-BMI	0.01	(−0.01, 0.03)	0.271	**0.02**	**(<0.01, 0.05)**	**0.040**	0.01	(−0.02, 0.04)	0.648	0.01	(−0.02, 0.03)	0.705
Model 2^b^												
TG (mg/dL)	0.07	(−0.05, 0.18)	0.250	0.06	(−0.05, 0.17)	0.282	0.02	(−0.12, 0.16)	0.798	0.07	(−0.06, 0.20)	0.269
HDLC (mg/dL)	−0.02	(−0.06, 0.03)	0.479	0.03	(−0.02, 0.08)	0.189	0.03	(−0.03, 0.10)	0.258	0.02	(−0.04, 0.07)	0.546
LDLC (mg/dL)	0.03	(−0.03, 0.09)	0.347	**0.11**	**(0.05, 0.17)**	**0.001**	0.07	(−0.01, 0.15)	0.075	**0.08**	**(<0.01, 0.15)**	**0.048**
TC (mg/dL)	0.02	(−0.02, 0.07)	0.259	**0.07**	**(0.02, 0.11)**	**0.002**	0.04	(−0.02, 0.09)	0.157	0.04	(> − 0.01, 0.09)	0.075
CRI-I	0.04	(−0.01, 0.09)	0.119	0.03	(−0.02, 0.09)	0.209	<0.01	(−0.06, 0.07)	0.906	0.03	(−0.03, 0.09)	0.374
CRI-II	0.05	(−0.03, 0.12)	0.202	**0.08**	**(0.01, 0.15)**	**0.034**	0.04	(−0.05, 0.13)	0.417	0.06	(−0.03, 0.14)	0.187
NHC	0.04	(−0.02, 0.10)	0.175	**0.08**	**(0.03, 0.14)**	**0.004**	0.04	(−0.03, 0.11)	0.273	0.06	(−0.01, 0.13)	0.094
AC	0.06	(−0.02, 0.13)	0.139	0.05	(−0.02, 0.13)	0.178	0.01	(−0.09, 0.10)	0.899	0.04	(−0.05, 0.13)	0.369
TyG-BMI	0.01	(−0.01, 0.03)	0.361	**0.03**	**(<0.01, 0.05)**	**0.018**	0.01	(−0.02, 0.04)	0.438	0.01	(−0.02, 0.04)	0.546

a Model 1: adjustment for age, sex, BMI, urinary creatinine levels, endocrine disease status, and Ln ΣDEHPm. Bold: *p* < 0.05.

b Model 2: adjustment for age, sex, education, income, PCPs use, BMI, urinary creatinine, endocrine disease status, and Ln ΣDEHPm. Bold: *p* < 0.05.

### 3.3 Associations between lipid metabolism and insulin resistance indices

Concerning the association between lipid metabolism and insulin resistance indices, TyG-BMI exhibited positive associations with TG (β = 3.02, *p* < 0.001), CRI-I (β = 0.98, *p* < 0.001), CRI-II (β = 1.03, *p* < 0.001), NHC (β = 0.63, *p* < 0.001), and AC (β = 1.07, *p* < 0.001). However, a negative association was observed between TyG-BMI and HDLC (Supplementary Table S1).

### 3.4 Mediating role of insulin resistance in the association between urinary paraben levels and lipid metabolism

**Figure 2:**
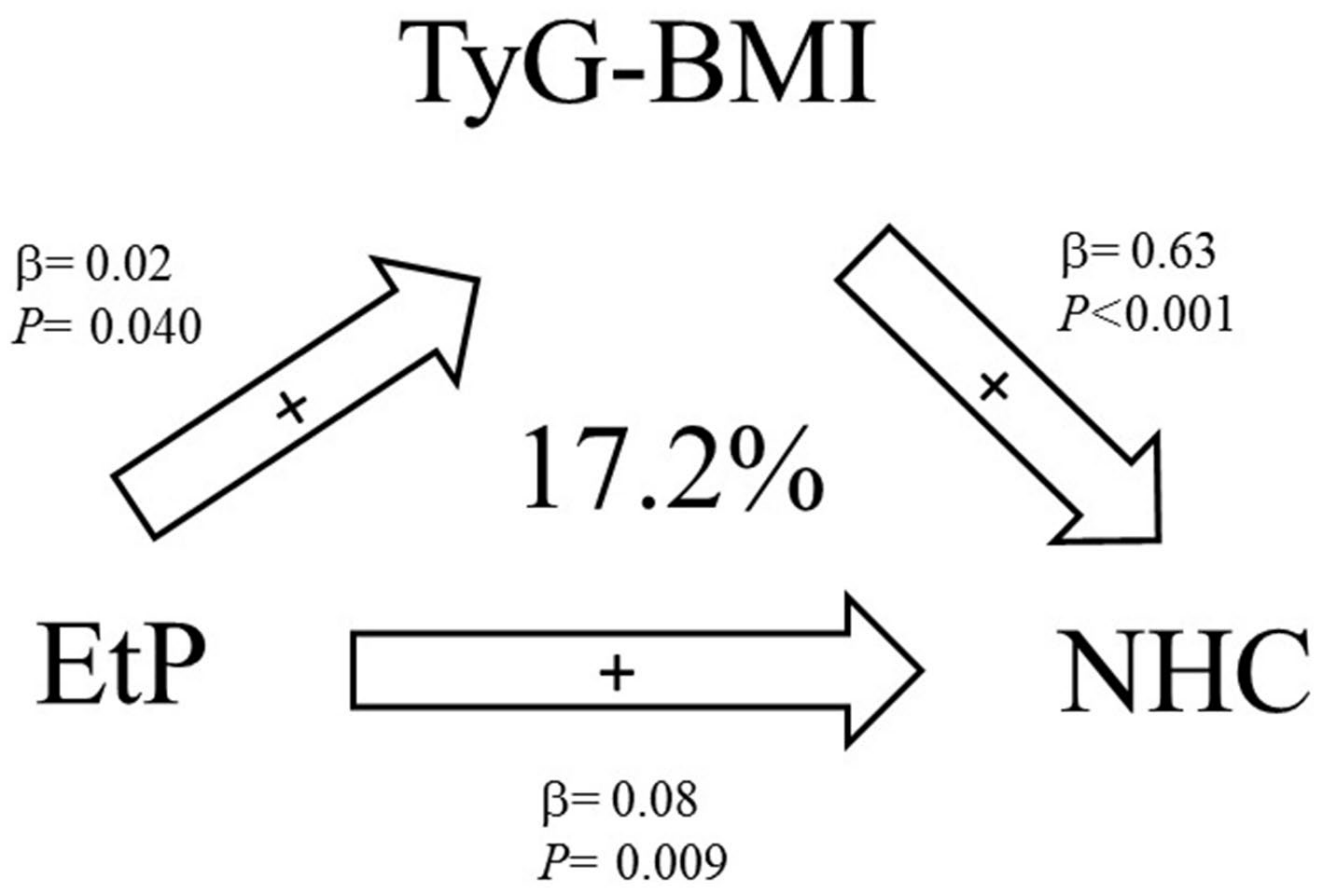
Mediation effects of exposure to parabens on the homeostatic model assessment of estimated lipid metabolism indices through insulin resistance indices (*N* = 264).

We selected parabens that, upon exposure, were significantly associated with lipid metabolism and insulin resistance indices to conduct a mediation analysis; in this analysis, we identified that insulin resistance could serve as mediators for the effects of paraben exposure on lipid metabolism indices. In mediation analysis, TyG-BMI mediated 17.2% of the association between EtP and NHC (indirect effect = 0.014, 95% confidence interval [CI] = 0.003–0.029); the mediation effect was significant (shown in Supplementary Table S2 and Figure 2).

## 4 Discussion

Our study revealed that MeP was positively associated with CRI-I and that EtP was positively associated with LDLC, TC, and NHC. The findings of this study indicate that exposure to parabens may be associated with obesity and indicators of lipid metabolism, as well as adverse health outcomes, in adults. These findings are consistent with those reported in previous epidemiological studies. In an ongoing three-year cycle cross-sectional biomonitoring programme [KoNEHS Cycle 3 (2015–2017)], an adult population (aged 19 years or older) was investigated to ascertain the current level of exposure to major environmental chemicals among the general Korean population. The KoNEHS study also revealed a positive association between urinary EtP levels in adults and obesity [β = 0.03, *p* = 0.038; (17)]. A total of 1,454 children, 891 adolescents, and 3,758 adults (for BMI) and 3,424 adults (for TG/HDL) from the Korean National Environmental Health Survey (2015 to 2017) were included in this cross-sectional study. The findings of Kim’s study suggest that exposure to EDC mixtures is associated with elevated BMI and TG/HDL levels in both adolescents and adults. The association is more pronounced in adults than in adolescents. Moreover, adolescence may signify a critical period for EDC mixtures in terms of outcomes (41).

In a related study, blood plasma samples were collected from 27 healthy women at various points throughout their menstrual cycles in order to examine the potential correlation between paraben exposure and obesity (58). The plasma levels of methylparaben, as well as the sum of parabens, were found to be positively associated with plasma adipsin levels. Conversely, a negative correlation was observed between methylparaben levels and glucagon, leptin, and PAI-1. These inconsistencies in the impact of urinary paraben concentrations on lipid metabolism indices could be attributed to differences in study design or participant characteristics, including sex, age, or ethnicity.

It is hypothesised that paraben exposure may impact insulin sensitivity in human organs, thus providing an underlying mechanism that could explain the observed association. Hu et al. (15) found that parabens promote adipogenesis in 3 T3-L1 cells, contributing to obesity by disrupting lipid synthesis and decomposition via the PPARγ receptor. Their findings showed a significant positive association between EtP and TyG-BMI. Animal studies suggest that paraben exposure can damage pancreatic islet cells. For example, zebrafish embryos exhibited enlarged islet areas, abnormal shapes, and increased aberrant β-cells (42). Pereira-Fernandes et al. (43) demonstrated that parabens strongly bind to and activate PPARγ, a key regulator of insulin sensitivity. Our results showed a significant positive association between EtP and TyG-BMI, suggesting that paraben exposure may disrupt blood glucose regulation and increase insulin resistance risk. These findings align with the KoNEHS Cycle 3 study (2015–2017) in South Korea, which reported positive associations between MeP (OR = 1.68, 95% CI = 1.08–2.60) and EtP (OR = 2.74, 95% CI = 1.77–4.24) with diabetes (17). Similarly, Bai et al. (44) found a significant positive association between PrP and insulin resistance (OR = 1.72, 95% CI = 1.15–2.57) in NHANES (2009–2016). A case–control study from the Henan Rural Cohort Study, including 1,713 participants (880 with type 2 diabetes and 833 controls), used generalized linear regression models to assess the effects of parabens on T2DM and insulin resistance indicators (63). The study found a linear positive association between MeP or paraben mixtures and T2DM risk, while EtP and BuP showed a non-linear association, with moderate-to-high exposure levels contributing to T2DM development (63). The findings of this study demonstrated that exposure to MeP or paraben mixtures was found to have a linear positive association with the risk of T2DM. EtP and BuP demonstrated a non-linear association with insulin resistance, with moderate-high exposure levels contributing to the development of T2DM (63).

A prospective study of 1,087 pregnant women from a single tertiary medical center also shows that urinary EtP was associated with gestational DM, with risk ratios of 1.12, 1.11 and 1.70 for the second, third and highest quartiles, respectively (64). Furthermore, a case–control study of adults (*n* = 101) in Jeddah, Saudi Arabia during 2015–2016 also found that increased parabens (including MeP, EtP, and PrP) exposure could lead to an over six-fold increase in the risk of diabetes (65).

We found that insulin resistance indices were positively linked to LDLC and lipid metabolism markers but negatively associated with HDLC, suggesting a role in dyslipidemia and obesity. Previous studies indicate that insulin resistance and type 2 diabetes can elevate TG or reduce HDLC levels (45, 46). Insulin resistance may impair VLDL degradation, leading to increased VLDL synthesis (47). VLDL transports fat from the liver to tissues and converts to LDLC after unloading most of its fat (48, 49).

Consequently, insulin resistance may lead to elevated TG levels, resulting in hypertriglyceridemia. Insulin resistance also reduces the activity of lipoprotein lipase, a key mediator of VLDL clearance (50). This reduction in VLDL and LDLC uptake by the liver prolongs the duration of these lipoproteins in the plasma (22, 47). Gencer et al. (51) confirmed that insulin resistance in polycystic ovary syndrome (PCOS) is linked to fasting insulin, HOMA index, BMI, and right ovarian volume. In PCOS with Hashimoto’s thyroiditis (PCOS+HT), insulin resistance also correlates with fasting insulin, HOMA index, BMI, SHBG, and left ovarian volume. Among PCOS patients with insulin resistance, 37.5% had increased right ovarian volume, while left ovarian volume was elevated in 35.7% of those without insulin resistance and 68.8% of those with it. PCOS shares clinical similarities with certain thyroid diseases, particularly hypothyroidism and autoimmune thyroid diseases (AITDs) (52). Its coexistence with hyperthyroidism is rare, suggesting thyroid influence on PCOS through metabolism and immunity. Thyroid function affects insulin resistance, a key factor in PCOS, with hypothyroidism exacerbating it more than hyperthyroidism. The rising prevalence of obesity further impacts health, as hypothyroid patients are prone to obesity, and those with both PCOS and hypothyroidism often have a higher BMI and greater metabolic disease risk. Further research is required to confirm these findings and to elucidate the underlying mechanisms. Nevertheless, it is evident that strategies to reduce EDC exposure from early life stages may be necessary to lower the risk of metabolic disease.

Our mediation analysis revealed that TyG-BMI could mediate the association between EtP and NHC. Therefore, TyG-BMI may be a mediator in the association between EtP exposure and NHC. Parabens increase the risk of obesity and cardiovascular disease by fostering the development of insulin resistance and dyslipidemia. Extensive epidemiological and mechanistic studies (both *in vivo* and *in vitro*) are warranted to validate these associations and elucidate the potential corresponding biological mechanisms.

There are four key strengths in this study. First, our current data were obtained from a representative survey including participants aged 7 to 97 years. Therefore, our study can accurately reflect the exposure profile of the general population in Taiwan. Second, few studies have explored the association between paraben exposure and metabolism indices in the general Taiwanese adult. Third, we employed various metabolic indices that are currently used in clinical practice but are rarely used in research, thus enriching the understanding of overall metabolic conditions. Finally, we conducted a mediation analysis to explore the potential mediating role of insulin resistance in the association between paraben exposure and lipid metabolism in the general Taiwanese adult population.

Despite its strengths, our study has some limitations that warrant consideration. First, we applied a cross-sectional design; hence, we could not establish a causal relationship between exposure and health effects. Second, our sample size was relatively small, which could potentially affect the reliability and interpretability of our statistical findings. Future research should consider a larger sample size for improved representativeness and robustness. Third, the measurement of urinary paraben concentrations using morning urine samples may not fully capture long-term exposure; however, this limitation is mitigated by evidence suggesting that daily exposure patterns for parabens could be consistent over time (53, 54).

## 5 Conclusion

The present study has revealed that parabens have the capacity to affect metabolic homeostasis. The potential mediation of the association between paraben exposure and lipid metabolism by insulin resistance is also indicated. The risk of obesity and cardiovascular disease is increased by parabens via the fostering of the development of insulin resistance and dyslipidemia. Whilst the participants in the present study were selected from the general population, the findings are limited to Taiwanese individuals. Therefore, future studies must include a greater number of samples in order to elucidate these underlying mechanisms and increase the generalizability of the results.

## Data Availability

The raw data supporting the conclusions of this article will be made available by the authors, without undue reservation.

## Supplementary material

### SUPPLEMENTARY FIGURE S1

Flow chart of the recruitment for the study.

### SUPPLEMENTARY FIGURE S2

Visualized directed acyclic graph (DAG) to apply the minimal sufficient adaptation set as covariates.

## References

[1] Lind PM Lind L. Endocrine-disrupting chemicals and risk of diabetes: an evidence-based review. Diabetologia. (2018) 61:1495–502. doi: 10.1007/s00125-018-4621-3, PMID: 29744538 PMC6445457

[2] Mottillo S Filion KB Genest J Joseph L Pilote L Poirier P. The metabolic syndrome and cardiovascular risk a systematic review and meta-analysis. J Am Coll Cardiol. (2010) 56:1113–32. doi: 10.1016/j.jacc.2010.05.034, PMID: 20863953

[3] Alberti KG Eckel RH Grundy SM Zimmet PZ Cleeman JI Donato KA. Harmonizing the metabolic syndrome: a joint interim statement of the international diabetes federation task force on epidemiology and prevention; National Heart, Lung, and Blood Institute; American Heart Association; world heart federation; international atherosclerosis society; and International Association for the Study of obesity. Circulation. (2009) 120:1640–5. doi: 10.1161/circulationaha.109.192644, PMID: 19805654

[4] Moller DE Kaufman KD. Metabolic syndrome: a clinical and molecular perspective. Annu Rev Med. (2005) 56:45–62. doi: 10.1146/annurev.med.56.082103.104751, PMID: 15660501

[5] Darbre PD. Endocrine disruptors and obesity. Curr Obes Rep. (2017) 6:18–27. doi: 10.1007/s13679-017-0240-4, PMID: 28205155 PMC5359373

[6] Mimoto MS Nadal A Sargis RM. Polluted pathways: mechanisms of metabolic disruption by endocrine disrupting chemicals. Curr Environ Health Rep. (2017) 4:208–22. doi: 10.1007/s40572-017-0137-0, PMID: 28432637 PMC5921937

[7] Song Y Chou EL Baecker A You NC Song Y Sun Q. Endocrine-disrupting chemicals, risk of type 2 diabetes, and diabetes-related metabolic traits: a systematic review and meta-analysis. J Diabetes. (2016) 8:516–32. doi: 10.1111/1753-0407.12325, PMID: 26119400

[8] Myridakis A Fthenou E Balaska E Vakinti M Kogevinas M Stephanou EG. Phthalate esters, parabens and bisphenol-a exposure among mothers and their children in Greece (Rhea cohort). Environ Int. (2015) 83:1–10. doi: 10.1016/j.envint.2015.05.014, PMID: 26072145

[9] Soni MG Carabin IG Burdock GA. Safety assessment of esters of p-hydroxybenzoic acid (parabens). Food Chem Toxicol. (2005) 43:985–1015. doi: 10.1016/j.fct.2005.01.020, PMID: 15833376

[10] Wei F Mortimer M Cheng H Sang N Guo LH. Parabens as chemicals of emerging concern in the environment and humans: a review. Sci Total Environ. (2021) 778:146150. doi: 10.1016/j.scitotenv.2021.146150, PMID: 34030374

[11] Haman C Dauchy X Rosin C Munoz JF. Occurrence, fate and behavior of parabens in aquatic environments: a review. Water Res. (2015) 68:1–11. doi: 10.1016/j.watres.2014.09.030, PMID: 25462712

[12] Junaid M Wang Y Hamid N Deng S Li W-G Pei D-S. Prioritizing selected PPCPs on the basis of environmental and toxicogenetic concerns: a toxicity estimation to confirmation approach. J Hazard Mater. (2019) 380:120828. doi: 10.1016/j.jhazmat.2019.120828, PMID: 31301631

[13] Nowak K Jablonska E Ratajezak-Wrona W. Controversy around parabens: alternative strategies for preservative use in cosmetics and personal care products. Environ Res. (2021) 198:110488. doi: 10.1016/j.envres.2020.110488, PMID: 33221305

[14] Honda M Robinson M Kannan K. Parabens in human urine from several Asian countries, Greece, and the United States. Chemosphere. (2018) 201:13–9. doi: 10.1016/j.chemosphere.2018.02.165, PMID: 29510318

[15] Hu P Chen X Whitener RJ Boder ET Jones JO Porollo A. Effects of parabens on adipocyte differentiation. Toxicol Sci. (2013) 131:56–70. doi: 10.1093/toxsci/kfs262, PMID: 22956630 PMC3621350

[16] Hu P Overby H Heal E Wang S Chen J Shen CL. Methylparaben and butylparaben alter multipotent mesenchymal stem cell fates towards adipocyte lineage. Toxicol Appl Pharmacol. (2017) 329:48–57. doi: 10.1016/j.taap.2017.05.019, PMID: 28527915 PMC5528845

[17] Lee I Park YJ Kim MJ Kim S Choi S Park J. Associations of urinary concentrations of phthalate metabolites, bisphenol a, and parabens with obesity and diabetes mellitus in a Korean adult population: Korean National Environmental Health Survey (KoNEHS) 2015–2017. Environ Int. (2021) 146:106227. doi: 10.1016/j.envint.2020.106227 33152652

[18] Bhardwaj S Bhattacharjee J Bhatnagar M Tyagi S Delhi N. Atherogenic index of plasma, Castelli risk index and atherogenic coefficient-new parameters in assessing cardiovascular risk. Int J Pharm Biol Sci. (2013) 3:359–64. Available at: https://ijpbs.com/ijpbsadmin/upload/ijpbs_526938e855804.pdf

[19] Packard CJ Saito Y. Non-HDL cholesterol as a measure of atherosclerotic risk. J Atheroscler Thromb. (2004) 11:6–14. doi: 10.5551/jat.11.6, PMID: 15067193

[20] Salcedo-Cifuentes M Belalcazar S Acosta EY Medina-Murillo JJ. Conventional biomarkers for cardiovascular risks and their correlation with the Castelli risk index-indices and TG/HDL-c. Archivos de Medicina (Manizales). (2020) 20:11–22. doi: 10.30554/archmed.20.1.3534.2020

[21] Monnier L Colette C Percheron C Descomps B. Insulin, diabetes and cholesterol metabolism. C R Seances Soc Biol Fil. (1995) 189:919–31. PMID: 8673637

[22] Ormazabal V Nair S Elfeky O Aguayo C Salomon C Zuñiga FA. Association between insulin resistance and the development of cardiovascular disease. Cardiovasc Diabetol. (2018) 17:122. doi: 10.1186/s12933-018-0762-4, PMID: 30170598 PMC6119242

[23] Huang HB Cheng PK Siao CY Lo YC Chou WC Huang PC. Mediation effects of thyroid function in the associations between phthalate exposure and lipid metabolism in adults. Environ Health. (2022) 21:61. doi: 10.1186/s12940-022-00873-9, PMID: 35778735 PMC9248169

[24] Huang PC Chen HC Chou WC Lin HW Chang WT Chang JW. Cumulative risk assessment and exposure characteristics of parabens in the general Taiwanese using multiple hazard indices approaches. Sci Total Environ. (2022) 843:156821. doi: 10.1016/j.scitotenv.2022.156821, PMID: 35738379

[25] Huang H-B Siao C-Y Lo Y-TC Shih S-F Lu C-H Huang P-C. Mediation effects of thyroid function in the associations between phthalate exposure and insulin resistance in adults. Environ Pollut. (2021) 278:116799. doi: 10.1016/j.envpol.2021.116799, PMID: 33743268

[26] Aubert N Ameller T Legrand JJ. Systemic exposure to parabens: pharmacokinetics, tissue distribution, excretion balance and plasma metabolites of [14C]-methyl-, propyl- and butylparaben in rats after oral, topical or subcutaneous administration. Food Chem Toxicol. (2012) 50:445–54. doi: 10.1016/j.fct.2011.12.045, PMID: 22265941

[27] Wang L Kannan K. Alkyl protocatechuates as novel urinary biomarkers of exposure to p-hydroxybenzoic acid esters (parabens). Environ Int. (2013) 59:27–32. doi: 10.1016/j.envint.2013.05.001, PMID: 23747757

[28] Abbas S Greige-Gerges H Karam N Piet MH Netter P Magdalou J. Metabolism of parabens (4-hydroxybenzoic acid esters) by hepatic esterases and UDP-glucuronosyltransferases in man. Drug Metab Pharmacokinet. (2010) 25:568–77. doi: 10.2133/dmpk.dmpk-10-rg-013, PMID: 20930423

[29] Boberg J Taxvig C Christiansen S Hass U. Possible endocrine disrupting effects of parabens and their metabolites. Reprod Toxicol. (2010) 30:301–12. doi: 10.1016/j.reprotox.2010.03.011, PMID: 20381602

[30] Calafat AM Ye X Wong LY Bishop AM Needham LL. Urinary concentrations of four parabens in the U.S. population: NHANES 2005-2006. Environ Health Perspect. (2010) 118:679–85. doi: 10.1289/ehp.0901560, PMID: 20056562 PMC2866685

[31] Ma WL Wang L Guo Y Liu LY Qi H Zhu NZ. Urinary concentrations of parabens in Chinese young adults: implications for human exposure. Arch Environ Contam Toxicol. (2013) 65:611–8. doi: 10.1007/s00244-013-9924-2, PMID: 23744051

[32] Wang L Wu Y Zhang W Kannan K. Characteristic profiles of urinary p-hydroxybenzoic acid and its esters (parabens) in children and adults from the United States and China. Environ Sci Technol. (2013) 47:2069–76. doi: 10.1021/es304659r, PMID: 23343203

[33] Ye X Bishop AM Reidy JA Needham LL Calafat AM. Parabens as urinary biomarkers of exposure in humans. Environ Health Perspect. (2006) 114:1843–6. doi: 10.1289/ehp.9413, PMID: 17185273 PMC1764178

[34] Zhang H Quan Q Li X Sun W Zhu K Wang X. Occurrence of parabens and their metabolites in the paired urine and blood samples from Chinese university students: implications on human exposure. Environ Res. (2020) 183:109288. doi: 10.1016/j.envres.2020.109288, PMID: 32311914

[35] Chen HC Chang JW Sun YC Chang WT Huang PC. Determination of parabens, bisphenol a and its analogs, Triclosan, and Benzophenone-3 levels in human urine by isotope-dilution-UPLC-MS/MS method followed by supported liquid extraction. Toxics. (2022) 10:21. doi: 10.3390/toxics10010021, PMID: 35051063 PMC8781104

[36] European Medicines Agency, Committee for Medicinal Products for Human Use. Guideline on Bioanalytical Method Validation. London, UK: European Medicines Agency. (2011).

[37] Huang HB Pan WH Chang JW Chiang HC Guo YL Jaakkola JJ. Does exposure to phthalates influence thyroid function and growth hormone homeostasis? The Taiwan environmental survey for toxicants (TEST) 2013. Environ Res. (2017) 153:63–72. doi: 10.1016/j.envres.2016.11.014, PMID: 27907809

[38] Waits A Chen HC Kuo PL Wang CW Huang HB Chang WH. Urinary phthalate metabolites are associated with biomarkers of DNA damage and lipid peroxidation in pregnant women—Tainan birth cohort study (TBCS). Environ Res. (2020) 188:109863. doi: 10.1016/j.envres.2020.109863, PMID: 32846647

[39] Er LK Wu S Chou HH Hsu LA Teng MS Sun YC. Triglyceride glucose-body mass index is a simple and clinically useful surrogate marker for insulin resistance in nondiabetic individuals. PLoS One. (2016) 11:e0149731. doi: 10.1371/journal.pone.0149731, PMID: 26930652 PMC4773118

[40] Lee I Kim S Park S Mok S Jeong Y Moon H-B. Association of urinary phthalate metabolites and phenolics with adipokines and insulin resistance related markers among women of reproductive age. Sci Total Environ. (2019) 688:1319–26. doi: 10.1016/j.scitotenv.2019.06.125, PMID: 31726561

[41] Kim J Chevrier J. Exposure to parabens and prevalence of obesity and metabolic syndrome: an analysis of the Canadian health measures survey. Sci Total Environ. (2020) 713:135116. doi: 10.1016/j.scitotenv.2019.135116, PMID: 32019002

[42] Brown SE Sant KE Fleischman SM Venezia O Roy MA Zhao L. Pancreatic beta cells are a sensitive target of embryonic exposure to butylparaben in zebrafish (*Danio rerio*). Birth Defects Res. (2018) 110:933–48. doi: 10.1002/bdr2.1215, PMID: 29516647 PMC6030486

[43] Pereira-Fernandes A Demaegdt H Vandermeiren K Hectors TL Jorens PG Blust R. Evaluation of a screening system for obesogenic compounds: screening of endocrine disrupting compounds and evaluation of the PPAR dependency of the effect. PLoS One. (2013) 8:e77481. doi: 10.1371/journal.pone.0077481, PMID: 24155963 PMC3796469

[44] Bai J Ma Y Zhao Y Yang D Mubarik S Yu C. Mixed exposure to phenol, parabens, pesticides, and phthalates and insulin resistance in NHANES: a mixture approach. Sci Total Environ. (2022) 851:158218. doi: 10.1016/j.scitotenv.2022.158218, PMID: 36028038

[45] Liu H Liu J Liu J Xin S Lyu Z Fu X. Triglyceride to high-density lipoprotein cholesterol (TG/HDL-C) ratio, a simple but effective Indicator in predicting type 2 diabetes mellitus in older adults. Front Endocrinol (Lausanne). (2022) 13:828581. doi: 10.3389/fendo.2022.828581, PMID: 35282431 PMC8907657

[46] Liu H Yan S Chen G Li B Zhao L Wang Y. Association of the Ratio of triglycerides to high-density lipoprotein cholesterol levels with the risk of type 2 diabetes: a retrospective cohort study in Beijing. J Diabetes Res. (2021) 2021:5524728–8. doi: 10.1155/2021/5524728, PMID: 33969127 PMC8081643

[47] Vergès B. Pathophysiology of diabetic dyslipidaemia: where are we? Diabetologia. (2015) 58:886–99. doi: 10.1007/s00125-015-3525-8, PMID: 25725623 PMC4392164

[48] Gershkovich P Hoffman A. Effect of a high-fat meal on absorption and disposition of lipophilic compounds: the importance of degree of association with triglyceride-rich lipoproteins. Eur J Pharm Sci. (2007) 32:24–32. doi: 10.1016/j.ejps.2007.05.109, PMID: 17604610

[49] Lambert D Mourot J. Vitamin E and lipoproteins in hyperlipoproteinemia. Atherosclerosis. (1984) 53:327–30. doi: 10.1016/0021-9150(84)90133-3 6529449

[50] Semenkovich CF. Insulin resistance and atherosclerosis. J Clin Invest. (2006) 116:1813–22. doi: 10.1172/JCI29024, PMID: 16823479 PMC1483180

[51] Gencer G Serin AN Gencer K. Analysis of the effect of hashimoto's thyroiditis and insulin resistance on ovarian volume in patients with polycystic ovary syndrome. BMC Womens Health. (2023) 23:86. doi: 10.1186/s12905-023-02200-x, PMID: 36829146 PMC9960704

[52] Fan H Ren Q Sheng Z Deng G Li L. The role of the thyroid in polycystic ovary syndrome. Front Endocrinol. (2023) 14:1242050. doi: 10.3389/fendo.2023.1242050, PMID: 37867519 PMC10585146

[53] Pollack AZ Perkins NJ Sjaarda L Mumford SL Kannan K Philippat C. Variability and exposure classification of urinary phenol and paraben metabolite concentrations in reproductive-aged women. Environ Res. (2016) 151:513–20. doi: 10.1016/j.envres.2016.08.016, PMID: 27567355 PMC5071150

[54] Smith KW Braun JM Williams PL Ehrlich S Correia KF Calafat AM. Predictors and variability of urinary paraben concentrations in men and women, including before and during pregnancy. Environ Health Perspect. (2012) 120:1538–43. doi: 10.1289/ehp.1104614, PMID: 22721761 PMC3556607

[55] Wong TJ Yu T. Trends in the distribution of body mass index, waist circumference and prevalence of obesity among Taiwanese adults, 1993-2016. PLoS One. (2022) 17:e0274134. doi: 10.1371/journal.pone.0274134 36084122 PMC9462812

[56] Pan W-H. In: Nutrition and Health Survey in Taiwan 2017–2020: Preliminary Results. Health Promotion Administration, Ministry of Health and Welfare, editor. Health Promotion Administration, Ministry of Health and Welfare; Taipei, Taiwan. (2020).

[57] Salamanca-Fernández E Iribarne-Durán LM Rodríguez-Barranco M Vela-Soria F Olea N Sánchez-Pérez MJ. Historical exposure to non-persistent environmental pollutants and risk of type 2 diabetes in a Spanish sub-cohort from the European Prospective Investigation into Cancer and Nutrition study. Environ Res. (2020) 185:109383. doi: 10.1016/j.envres.2020.109383 32224340

[58] Kolatorova L Sramkova M Vitku J Vcelak J Lischkova O Starka L. Parabens and their relation to obesity. Physiol Res. (2018) 67:S465–S472. doi: 10.33549/physiolres.934004 30484673

[59] Health Promotion Administration, Ministry of Health and Welfare. Taiwan’s Obesity Prevention and Management Strategy. 1st edn, 1:55. (Health Promotion Administration, Ministry of Health and Welfare), (2018).

[60] Lohman TG Roche AF Martorell R. Anthropometric Standardization Reference Manual. Human Kinetics; Champaign, IL, USA. (1988).

[61] Textor J van der Zander B Gilthorpe MS Li´skiewicz M Ellison GT. Robust causal inference using directed acyclic graphs: The R package ‘dagitty’. Int J Epidemiol. (2016) 45:1887–94. 28089956 10.1093/ije/dyw341

[62] VanderWeele TJ. Explanation in Causal Inference: Methods for Mediation and Interaction. (2015) New York: Oxford University Press.

[63] Song Y Wang M Nie L Liao W Wei D Wang L. Exposure to parabens and dysglycemia: Insights from a Chinese population. Chemosphere. (2023) 340:139868. doi: 10.1016/j.chemosphere.2023.139868 37597620

[64] Liu W Zhou Y Li J Sun X Liu H Jiang Y. Parabens exposure in early pregnancy and gestational diabetes mellitus. Environ Int. (2019) 126:468–75. doi: 10.1016/j.envint.2019.02.040 30844582

[65] Li AJ Xue J Lin S Al-Malki AL Al-Ghamdi MA Kumosani TA. Urinary concentrations of environmental phenols and their association with type 2 diabetes in a population in Jeddah, Saudi Arabia. Environ Res. (2018) 166:544–52. doi: 10.1016/j.envres.2018.06.040 29960220

