# Supplementary material for: Mediating effects of insulin resistance on lipid metabolism with elevated paraben exposure in the general Taiwan population
Source: Front Public Health. 2025 Mar 14;13:1416264. doi: 10.3389/fpubh.2025.1416264 (PMC11949955; doi:10.3389/fpubh.2025.1416264)
Supplement: Supplementary file 1 [file Data_Sheet_1.docx]

**Supplements**

**Mediating Effects of Insulin Resistance on Lipid Metabolism with Elevated Paraben Exposure in the General Taiwan Population**

Po-Chin Huang ^a,b,c^, Hsin-Chang Chen ^d^, Han-Bin Huang ^e^, Yu-Lung Lin ^a^, Wan-Ting Chang ^a^, Shih-Hao Leung ^f^, Hsi Chen ^f^, Jung-Wei Chang ^f,*^

^a^ National Institute of Environmental Health Sciences, National Health Research Institutes, Miaoli, Taiwan

^b^ Department of Medical Research, China Medical University Hospital, China Medical University, Taichung, Taiwan

^c^ Research Center for Precision Environmental Medicine, Kaohsiung Medical University, Kaohsiung, Taiwan

^d^ Department of Chemistry, Tunghai University, Taichung, Taiwan;

^e^ School of Public Health, National Defense Medical Center, Taipei, Taiwan

^f^ Institute of Environmental and Occupational Health Sciences, School of Medicine, National Yang-Ming University, Taipei, Taiwan

**Table S1.** Adjusted regression coefficient (β), 95% confidence intervals (CI), and p-values (p) for change in lipid metabolism indicators in relation to unit-increased in insulin resistance in Taiwanese adults (N=264).

| Variables | TyG-BMI | | |
| --- | --- | --- | --- |
|  | **β** | **95% CI** | ***p* value** |
| TG (mg/dL) | **3.02** | **(2.58, 3.47)** | **<0.001** |
| HDLC (mg/dL) | **-0.79** | **(-1.02, -0.57)** | **<0.001** |
| LDLC (mg/dL) | 0.23 | (-0.10, 0.56) | 0.167 |
| TC (mg/dL) | 0.18 | (-0.04, 0.40) | 0.100 |
| CRI-I | **0.98** | **(0.73, 1.23)** | **<0.001** |
| CRI-II | **1.03** | **(0.66, 1.39)** | **<0.001** |
| NHC | **0.63** | **(0.34, 0.92)** | **<0.001** |
| AC | **1.43** | **(1.07, 1.78)** | **<0.001** |

TG= Triglycerides, HDLC= High Density Lipoprotein Cholesterol, LDLC= Low Density Lipoprotein Cholesterol, and TC= Total cholesterol; Bold: *p*<0.05

**Table S2.** Mediation effects of exposure to parabens on the homeostatic model assessment of estimated lipid metabolism indicators through insulin resistance indicators ^a^ (N=264).

| Exposure and outcome | Mediator | Estimate direct effect (95% CI) | Estimate indirect effect (95% CI) | Estimated proportion mediated |
| --- | --- | --- | --- | --- |
| EtP & NHC ^b^ | TyG-BMI ^b^ | **0.067 (0.012, 0.121)**^*^ | **0.014 (0.003, 0.029)** ^*^ | 17.2% |

*p*<0.05.

^a^ Adjustment for age, sex, BMI, urinary creatinine levels, endocrine disease status, and Ln ΣDEHPm.

^b^ EtP, TyG-BMI and NHC were natural log-transformed.

**Recruitment exceptions**

- Pregnant
- Breast-feeding women
- Severe illness (e.g., cancer)
- Foreign nationals
- Imprisoned or hospitalized

Samples collected between May 2013 to December 2013. (N=394)

**Excluded**

- 28 participants with insufficient urinary samples
- 27 participants with insufficient biochemical indicators
- 75 minors

264 participants in final list

(Age: 18 ≥ yrs)

**Questionnaire**

**Urinary & Blood**

1. **Parabens:**

MeP, EtP, PrP, BuP

1. **Metabolism indicators:**

fasting glucose, fasting insulin, Triglycerides, High-density lipoprotein (HDL), Low-Density Lipoprotein Cholesterol (LDL), Total Cholesterol (TC)

1. **Demographic characteristics**
2. **Lifestyle, dietary and PCP usage habit**
3. **Personal disease history**

(e.g., Cardiovascular, respiratory, urinary, digestive, reproductive system, endocrine disease and cancer)

**Fig S1.**


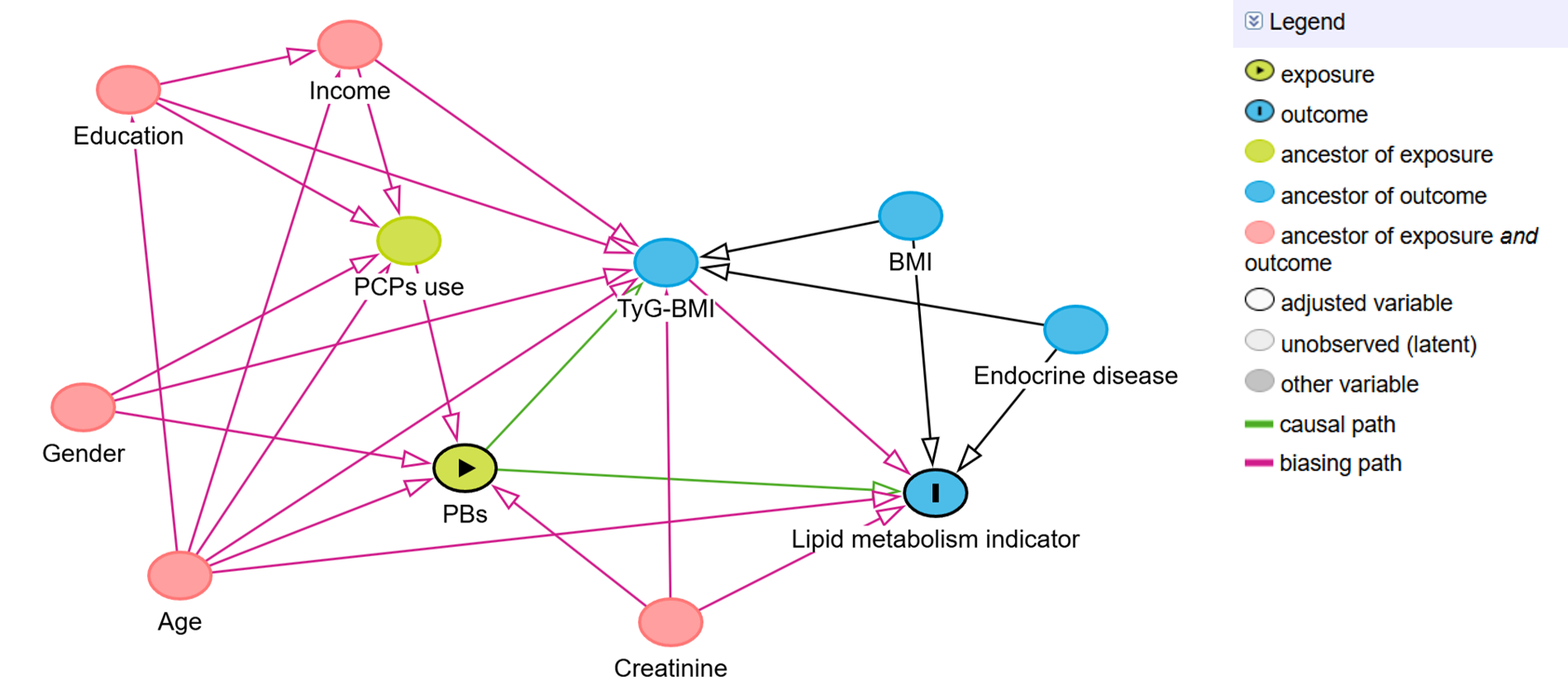


**Fig S2.**
